# Can indoor plants reduce formaldehyde levels in the anatomy dissection hall? A study to evaluate the practicality of using plants in reducing formaldehyde levels

**DOI:** 10.12688/f1000research.152489.1

**Published:** 2024-10-18

**Authors:** Adish G Joshi, Dhiren Punja, Rohini Punja

**Affiliations:** 1 Kasturba Medical College, Manipal, Manipal Academy of Higher Education, Manipal, Karnataka, 576104, India

**Keywords:** Air quality; anatomical dissection hall, indoor plants, indoor air, volatile organic compound, workspace environment, cadaver, formaldehyde

## Abstract

**Background:**

Formaldehyde a commonly used embalming fluid for the preservation of cadavers, produces numerous short and long-term side effects on the students and staff working with it. Indoor plants have been shown to reduce atmospheric formaldehyde levels. The purpose of the study was to compare the efficacy of indoor plants in reducing formaldehyde levels in ambient air to levels deemed safe by WHO standards

**Methods:**

The study was conducted in the storage room of the Anatomical dissection hall. 7 potted plants of one variety were kept in the storage room for 14 days and the Formaldehyde reading was measured using a VOC (Volatile Organic Compound) monitor. Then the same was done with no plant. This cycle was repeated 2 more times, with two different plant species. The three spieces of plants used for this study were as follows: *Dracaena trifasciata*, *Epipremnum aureum*, *Spathiphyllum.*

**Results:**

The results were analysed and compared and it was determined that the effect of having a plant present versus having no plant present on the Formaldehyde concentration in the air was inconclusive as *Dracaena trifasciata* failed to reject the null hypothesis altogether (p-value>0.05), while *Epipremnum aureum* and *Spathiphyllum* rejected the null hypothesis (p-value<0.05), however they both had a weak positive correlation with formaldehyde concentration.

**Conclusion:**

The efficacy of indoor plants in reducing formaldehyde levels in ambient air needs to be further explored and validated since all the prior studies conducted were in controlled environment and should be done in a real time scenario for its practical and beneficial uses.

## Introduction

Formalin is an ideal embalming fluid used in the preservation of the human body since it acts as a biocide by coagulating the bacterial protoplasm and is a powerful germicide. It preserves tissues by making new complex molecules that are unfit for the growth of microorganisms.

Medical students who are exposed to formaldehyde (FA) during their dissection course ( Shiraishi 2006) have reported various physical symptoms, such as burning eyes, lacrimation, irritation of airways, and dermatitis. FA has attracted attention as a health hazard for students and instructors, as FA concentrations in the air of gross anatomy laboratories often exceed permissible limits. On an average, students and instructors are exposed to 5.59 ppm FA, for which the maximum permissible exposure limit, according to OSHA PEL guidelines, is 0.75 ppm—essentially more than 7 times the maximum amount—and is more than 69 times greater than the recommended levels of FA exposure in the short-term and long-term (0.08 ppm) according to the Indoor Air Quality Guidelines set by the WHO ( Zuber et al. 2022; CDC n.d.; Nielsen et al. 2017). Long-term FA exposure in indoor air has been found to be carcinogenic, potentially leading to nasopharyngeal carcinoma and leukemia ( Shiraishi 2006; Zuber et al. 2022; CDC n.d.; Nielsen et al. 2017). High-quality indoor air cleaners can be utilized to reduce FA levels in the air with various types of air cleaners, such as activated carbon, negative ion and photocatalytic cleaners; however, these cleaners consume vast amount of electrical power (between 39.1 W and 79.2 W), can cause secondary FA pollution, are very expensive (between $147 and $721), and are inadequate for reducing FA concentrations to meet WHO guidelines. This makes them ineffective to use in an anatomy dissection hall ( Chen et al. 2007). Students and faculty utilize N95 masks as a precautionary method against the COVID-19 pandemic and wear them even in the anatomy dissection hall; however, N95 masks seem to be ineffectual at reducing FA levels to WHO standards, and hence, they fail to provide any additional benefit against the effects of FA ( Chan et al. 2016). Plants affect the levels of volatile organic compounds (VOCs) in indoor environments; thus, they represent a potentially cheaper, non-electricity-consuming and greener solution for improving indoor air quality ( Shaham et al. 2003). Studies of plants that reduce indoor pollution have recommended that 15 to 18 plants in 6 to 8-inch-diameter containers are required to clean the air in an average of 1,800 square foot houses. There is approximately one plant per 100 square feet of floor space ( Dela Cruz et al. 2014). Studies have indicated that the average FA absorption rate of certain plants is 674 micrograms per hour for *Spathiphyllum* (peace lily) and 1304 micrograms per hour for *Dracaena trifasciata/Sansevieria* (variegated snake plant) ( Wolverton and Wolverton 1993). The FA concentration in the atmosphere is affected by and directly proportional to the temperature and humidity of the air, the correlation coefficient between temperature and emission factors is greater than 0.83, and the correlation coefficient between relative humidity and emission factors is greater than 0.98 ( Parthasarathy et al. 2011).

Previous studies have mentioned the usage of potted ornamental plants in the reduction of FA pollution in indoor settings, and the deleterious effects of FA on individuals, especially medical students and teaching faculty in the anatomy dissection hall, no study thus far has dealt with uniting the two, by utilizing a cheap, effective and green way of reducing FA concentration in the anatomy dissection hall.

## Methods

### Ethical approval

The protocol was approved by the Institutional Ethics Committee (IEC 343/2022) Kasturba Medical College and Kasturba Hospitals, Manipal on July 14, 2022. Though the study did not involve cadavers directly, however the specimen storage room was utilized and written informed consent was given by the body donors for teaching and research when they were alive.

### Study design and data collection

**Figure 1.**
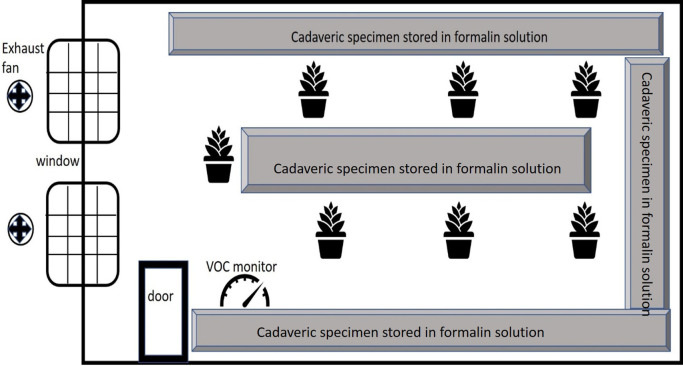
Diagram depicting the storage room with the position of the plants, the cadaveric specimen stored in formalin and the ventilation of the room.

The study was conducted in the cadaveric specimen storage room in the Anatomy Department, Kasturba Medical College, Manipal. The traffic into the storage room was restricted during the study period to minimize errors in recording FA levels. The storage room of the anatomy dissection hall has an area of 700 sq feet with two open windows, which were kept closed at all times, and two exhaust fans, which were not switched on during the study period. The VOC monitor installed in this room was initially used to assess the basal levels of FA fumes in the room. The values were recorded twice daily (at 9:00am and 4:00pm) for two weeks. Following which, 7 potted plants all of a particular plant species were kept in the room. Hence, there was one potted plant for every 100 sq. feet. The three species of plants used for this study were *Dracaena trifasciata/Sansevieria* (snake plant), *Epipremnum aureum* (golden pothos), *Spathiphyllum* (peace lily). The plants were in uniform pots with a frustum shape, with a top circumference of 79 cm and a bottom diameter of 44 cm. The height of the pot was 25 cm. The pots were kept equidistant from each other at a distance of 25 cm. However, the dimensions of the plants per se were variable. All the potted plants were placed around the cadaveric specimen storage room as seen in Figure 1. The plants were kept here for 2 weeks and the FA values were recorded twice daily. These potted plants received natural indirect sunlight for ten hours. This study was conducted during the summer season, and the temperature and humidity were noted along with the FA levels. The plants were subsequently removed, and a two-week interval was established before the plants of the next species were transferred to allow the FA concentrations to once again reach basal levels. The daily temperature and humidity were also noted.

### Data analysis

The readings documented were tabulated, and the statistical analysis was done using IBM SPSS Statistics for Windows Version 20.0 (USA). The P value for each of the plant species was calculated separately to determine whether any of the plants had a statistically significant effect on the FA concentration in the storage room. The P value ranges from 0 to 1, where a value closer to 0 means that there is a good chance of statistical significance, while a value closer to 1 indicates a null hypothesis; i.e., there is no statistical significance.

## Results

**Figure 2.**
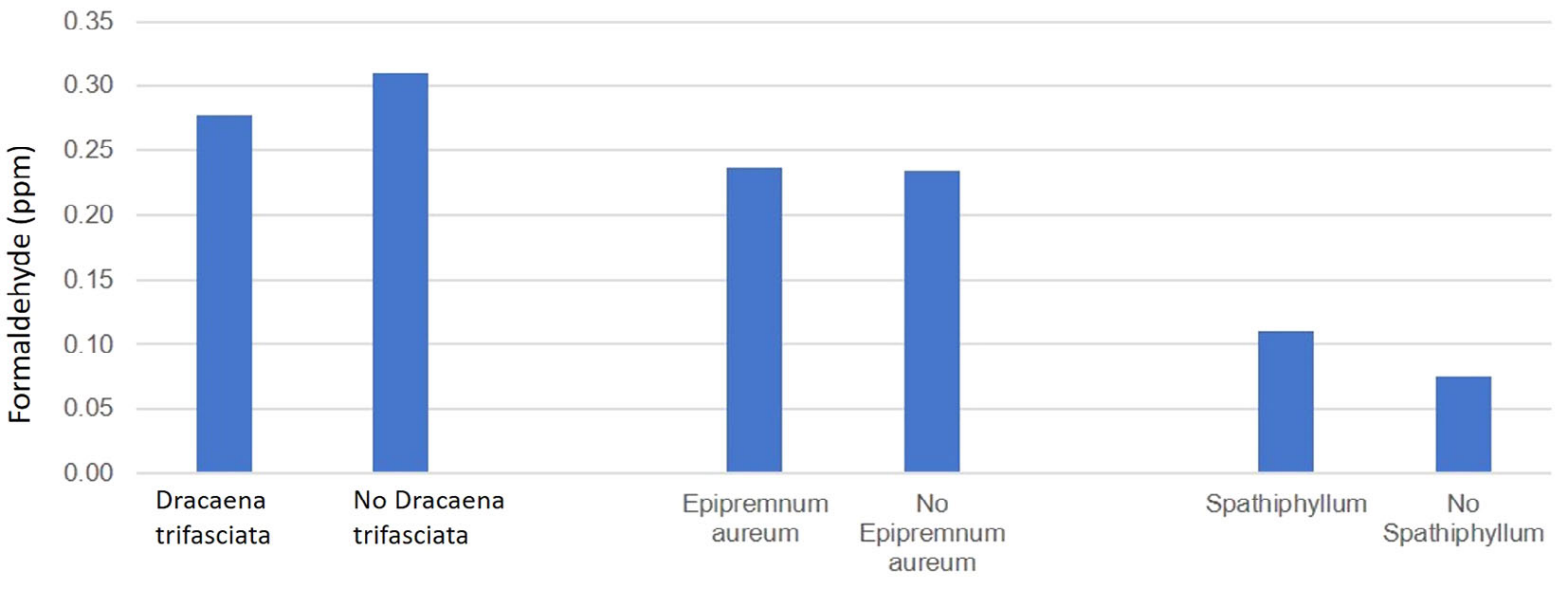
Efficacy of 3 plant species in reducing FA levels in the storage room of the anatomy dissection hall.

On observing Figure 2, plotted with the plants across the x-axis and concentration of FA across the y-axis and values from Table 1, the following inference could be made regarding the FA concentration measured in the storage room:

1. When *Dracaena trifasciata* is present, the concentration in the room was 0.03 ppm less than when there is an absence of the plant.
2. When *Epipremnum aureum* was present, the concentration in the room was 0.01 ppm greater than that when no *Epipremnum aureum* was present.
3. When *Spathiphyllum* is present, the room has 0.03 ppm more than when no *Spathiphyllum* is present.

**Table 1.** The average temperature, humidity and FA concentrations with/without the plants.

	No plant			With plant		
	Avg. Temperature (°C)	Avg. Humidity	Avg. Formaldehyde (ppm)	Avg. Temperature (°C)	Avg. Humidity	Avg. Formaldehyde (ppm)
1. *Dracaena trifasciata*	33.4	69%	0.31	32.8	69%	0.28
2. *Epipremnum aureum*	31.4	67%	0.23	32.5	72%	0.24
3. *Spathiphyllum*	27.0	82%	0.08	26.3	85%	0.11

Hence, according to the results obtained, *Dracaena trifasciata* had the greatest negative effect on FA levels and helped to reduce FA concentration, while *Epipremnum aureum* and *Spathiphyllum* had a comparatively different effect in its efficacy of reducing FA levels.

The P value for *Dracaena trifasciata* was 0.06, that for *Epipremnum aureum* was 0.02, and that for *Spathiphyllum* was <0.01.

The regressions of *Epipremnum aureum* and *Spathiphyllum* were calculated, and both had nonsignificant increase in formaldehyde levels in the air compared with the absence of plants in the storage room.

Hence, *Dracaena trifasciata* fails to reject/invalidate the null hypothesis, while *Epipremnum aureum* and *Spathiphyllum* reject/invalidate the null hypothesis but have a slight positive effect on the FA level, there was an increase in the FA levels in the storage room, which is contrary to what we were trying to establish.

## Discussion

Earlier studies conducted by multiple authors such as Teiri et al. (2018), Dela Cruz et al. (2014), Kim et al. (2008) and Wolverton and Wolverton (1993) have shown that potted ornamental plants seem to have a significant negative correlation with the FA levels. *Spathiphyllum* and *Dracaena trifasciata* in question have been shown to reduce FA significantly ( Wolverton and Wolverton 1993); however, these same results were not reciprocated in this particular study. We did find a decrease in FA levels when *Dracaena trifasciata* was kept in the storage room; however, this change was not the same for the other 2 species of plants. While the efficacy of the *Epipremnum aureum* has not been particularly accurately described or reviewed in the literature, it is expected to reduce FA as well. All 3 plants failed to bring the average FA concentration in the storage room to levels deemed safe by WHO standards. However, the average FA levels in the storage room, whether there were any of the 3 plants or no plants at all, satisfied the OSHA standards. A similar result was also observed in previous studies ( Gahukar et al. 2014).

Temperature and humidity have a positive correlation with FA concentration, as observed in earlier literature ( Parthasarathy et al. 2011), which indicates a similar positive correlation. The efficacy of the indoor plants *Spathiphyllum*, *Dracaena trifasciata* and *Epipremnum aureum* in reducing FA levels in ambient air contradicts the findings of earlier studies and can be explained on the basis of the following hypothesis. In previous studies ( Teiri et al. 2018; Dela Cruz et al. 2014; Kim et al. 2008; Wolverton and Wolverton 1993), a constant amount of FA was pumped into a chamber; however, in the present study the FA present in the storage room in the anatomy dissection hall was evaluated and we wanted to determine the true efficacy of using potted plants in a real-time scenario. Humidity and temperature are clearly positively correlated with FA concentration, both in this study and in earlier studies ( Parthasarathy et al. 2011). However, the effects of these compounds on plant FA absorption and metabolism have not been determined. Previous studies ( Dela Cruz et al. 2014) have indicated the use of 1 plant per 100 sq. feet, which is the rationale behind putting 7 plants in the 700 sq. feet storage room, the conditions in the earlier study (controlled environment with known, constant quantities of FA) and this particular study (a real-time environment) differed, and a greater number of plants could impact the results of the study in a radically different way.

Studies conducted for longer duration could yield different results, as potted ornamental plants become sensitized to FA and hence metabolize and remove more FA over time if they are constantly exposed to it ( Wolverton and Wolverton 1993).

Considering the ill effects which long term exposure to FA could lead to as an occupational hazard ( Onyeka et al. 2018); causing decreased pulmonary functions in faculty and medical students exposed to FA during anatomy dissection ( Homwutthiwong and Ongwandee 2017) we hope to bring about a change in the working environment naturally. We plan to continue this study bearing the confounding factors in mind, scaling up the number of potted plants and hope that we could get an ideal environment where FA concentrations would not affect our health and learning would continue as usual. Researchers can explore the potential beneficial effects of indoor plants on reducing atmospheric pollutants, namely, FA, by such cost-effective methods. By delving deeper into this field and obtaining an ideal number and variety of plants for economically improving the air quality, especially in places with maximum exposure to FA, we hope to bring a positive change to those exposed to FA.

## Conclusion

The efficacy of using indoor plants for reducing FA levels needs to be further explored since studies conducted in ambient air are rare, unlike prior studies conducted in controlled environments. This study provides a scope for improvement in the future where potted plants are kept for longer durations to study their long-term effects on FA concentrations in air and increase the number of potted plants utilized.

## Ethics and consent statement

The protocol was approved by the Institutional Ethics Committee (IEC 343/2022) Kasturba Medical College and Kasturba Hospitals, Manipal on July 14, 2022. Though the study did not involve cadavers directly, however the specimen storage room was utilized and written informed consent was given by the body donors for teaching and research when they were alive.

## Data Availability

Fig share: Can indoor plants reduce formaldehyde levels in the anatomy dissection hall? A study to evaluate the practicality of using plants in reducing formaldehyde levels. https://doi.org/10.6084/m9.figshare.25911100.v3 ( Joshi et al. 2024) This project contains the following underlying data:
- EX sheet – Master Sheet.xlsx Data are available under the terms of the Creative Commons Attribution 4.0 International license (CC-BY 4.0). Figshare: STROBE checklist ‘Can indoor plants reduce formaldehyde levels in the anatomy dissection hall? A study to evaluate the practicality of using plants in reducing formaldehyde levels. https://doi.org/10.6084/m9.figshare.25911100.v3 ( Joshi et al. 2024) Data are available under the terms of the Creative Commons Attribution 4.0 International license (CC-BY 4.0).
