# Supplementary material for: Can indoor plants reduce formaldehyde levels in the anatomy dissection hall? A study to evaluate the practicality of using plants in reducing formaldehyde levels
Source: F1000Res. 2025 Jul 22;13:1249. Originally published 2024 Oct 18. [Version 3] doi: 10.12688/f1000research.152489.3 (PMC11803571; doi:10.12688/f1000research.152489.3)
Supplement: Peer review history

# Reviewer report: Reviewer response for version 3

**DOI:** 10.5256/f1000research.185228.r399863

**Published:** 2025-08-05

**Authors:** Choy Ker Woon

**Affiliations:** 1 Department of Anatomy, Faculty of Medicine, Universiti Teknologi MARA, Shah Alam, Malaysia

I have made recommendation to accept this manuscript for indexing.

Is the work clearly and accurately presented and does it cite the current literature?

Yes

If applicable, is the statistical analysis and its interpretation appropriate?

Partly

Are all the source data underlying the results available to ensure full reproducibility?

Partly

Is the study design appropriate and is the work technically sound?

Yes

Are the conclusions drawn adequately supported by the results?

Yes

Are sufficient details of methods and analysis provided to allow replication by others?

Partly

Reviewer Expertise:

NA

I confirm that I have read this submission and believe that I have an appropriate level of expertise to confirm that it is of an acceptable scientific standard.

# Reviewer report: Reviewer response for version 2

**DOI:** 10.5256/f1000research.175209.r348114

**Published:** 2025-02-04

**Authors:** Choy Ker Woon

**Affiliations:** 1 Department of Anatomy, Faculty of Medicine, Universiti Teknologi MARA, Shah Alam, Malaysia

1) Is there any significant differences between control and experimental group? Include the p value and its significant symbols in the table. 

 2) Under data analysis, state the type of the test done.

 3) Is there any differences compare with or without the exhaust fans switched on, which mimic the real environment where the students are in?

Is the work clearly and accurately presented and does it cite the current literature?

Yes

If applicable, is the statistical analysis and its interpretation appropriate?

Partly

Are all the source data underlying the results available to ensure full reproducibility?

Partly

Is the study design appropriate and is the work technically sound?

Yes

Are the conclusions drawn adequately supported by the results?

Yes

Are sufficient details of methods and analysis provided to allow replication by others?

Partly

Reviewer Expertise:

comparative anatomy, forensic antheopology and medical education

I confirm that I have read this submission and believe that I have an appropriate level of expertise to confirm that it is of an acceptable scientific standard, however I have significant reservations, as outlined above.

# Response

**Published:** 2025-07-16

**Authors:** Rohini Punja

**Affiliations:** Anatomy, Manipal Academy of Higher Education, Manipal, Karnataka, India

Reviewer comment:

 Is there any significant differences between control and experimental group? Include the p value and its significant symbols in the table.

Response: We appreciate this suggestion and have now added the p-values directly to Table 1, along with significance symbols (* for p<0.05, ** for p<0.01) to improve clarity of statistical significance between control and experimental groups.

 Reviewer comment:

 Under data analysis, state the type of the test done.

**Response: We have now explicitly stated in the data analysis section that paired t-tests were used to compare formaldehyde levels with and without plants for each plant species.**

 Reviewer comment:

 Is there any differences compare with or without the exhaust fans switched on, which mimic the real environment where the students are in?

**Response: This is an excellent point. Our current study focused on establishing baseline measurements without ventilation, which is why the exhaust fans were switched off during the experiment. We have now addressed this limitation in the methodology and conclusion sections, noting that future studies should compare scenarios with exhaust fans operational versus non-operational to better simulate actual teaching conditions.**

# Reviewer report: Reviewer response for version 2

**DOI:** 10.5256/f1000research.175209.r348122

**Published:** 2025-01-03

**Authors:** Yasar Sajjad

**Affiliations:** 1 COMSATS University Islamabad, Islamabad, Islamabad Capital Territory, Pakistan

The idea to test the indoor plants for their removal efficiency in real environment is appealing. The experiment was conducted carefully, and the article is also well written. I am concerned about the reliability of technique used to measure the formaldehyde. Conventionally used VOCs meters are not so accurate, and sometimes may produce false positive/negative results.  I suggest to address this point for future study in the conclusion section as well. 

 The clarity in the few sections is also required as follows.

 Materials and methods: Mention the model of VOC (Volatile Organic Compound) monitor with its detection limit. Whether this monitor is specialized for formaldehyde only or for all general VOCs ?.

 Results: Need to add the SE/SD on bars in fig 2.

 Also add the results of regression in supplementary data file

Is the work clearly and accurately presented and does it cite the current literature?

Yes

If applicable, is the statistical analysis and its interpretation appropriate?

Partly

Are all the source data underlying the results available to ensure full reproducibility?

Yes

Is the study design appropriate and is the work technically sound?

Yes

Are the conclusions drawn adequately supported by the results?

Yes

Are sufficient details of methods and analysis provided to allow replication by others?

Partly

Reviewer Expertise:

Plant Stress physiology

I confirm that I have read this submission and believe that I have an appropriate level of expertise to confirm that it is of an acceptable scientific standard, however I have significant reservations, as outlined above.

# Response

**Published:** 2025-07-16

**Authors:** Rohini Punja

**Affiliations:** Anatomy, Manipal Academy of Higher Education, Manipal, Karnataka, India

Reviewer Comment:

 The idea to test the indoor plants for their removal efficiency in real environment is appealing. The experiment was conducted carefully, and the article is also well written. I am concerned about the reliability of technique used to measure the formaldehyde. Conventionally used VOCs meters are not so accurate, and sometimes may produce false positive/negative results.  I suggest to address this point for future study in the conclusion section as well.

**Response: We thank the reviewer for acknowledging the value of our real-environment testing approach. We agree with the concern about VOC meter reliability and have added information addressing this limitation in both the methodology and conclusion sections. We have now specifically mentioned this as a direction for future research in the conclusion.**

 Reviewer Comment on Material and Methods:

 Mention the model of VOC (Volatile Organic Compound) monitor with its detection limit. Whether this monitor is specialized for formaldehyde only or for all general VOCs ?.

**Response: We have now added this information in the methods section, specifying that we used a Forbix Semicon air quality monitor with a detection limit of 0.005 ppm (5 parts per million) ppm.**

 Reviewer Comment on Results:

 Need to add the SE/SD on bars in fig 2.

**Response: We have now added SE information in the fig2 .**

# Reviewer report: Reviewer response for version 2

**DOI:** 10.5256/f1000research.175209.r343897

**Published:** 2024-12-12

**Authors:** Kumar Satish Ravi

**Affiliations:** 1 All India Institute of Medical Sciences, Gorakhpur, Uttarakhand, India

Authors have addressed my queries. Article may be accepted.

Is the work clearly and accurately presented and does it cite the current literature?

Yes

If applicable, is the statistical analysis and its interpretation appropriate?

I cannot comment. A qualified statistician is required.

Are all the source data underlying the results available to ensure full reproducibility?

Yes

Is the study design appropriate and is the work technically sound?

Yes

Are the conclusions drawn adequately supported by the results?

Yes

Are sufficient details of methods and analysis provided to allow replication by others?

Yes

Reviewer Expertise:

Anatomy, Cytogentics, Medical Education, Neuroanatomy

I confirm that I have read this submission and believe that I have an appropriate level of expertise to confirm that it is of an acceptable scientific standard.

# Reviewer report: Reviewer response for version 1

**DOI:** 10.5256/f1000research.167255.r333503

**Published:** 2024-11-14

**Authors:** Ranganath Vallabhajosyula

**Affiliations:** 1 Nanyang Technological University Singapore, Singapore,, Singapore

The authors have conducted an interesting study on role of indoor plants in reducing the formaldehyde levels in the cadaveric labs.  Interesting though, there are few questions, that authors would need to address.

 Introduction:  The authors have brought interesting statements on exposure of the quantum of FA concentrations and its effects.  At this point, the authors need to emphasize on the composition of the formaldehyde to embalm the cadavers in general as well as experimental site.  

 Material and methods:  The authors have explained the size of the plants and arrangement of positions in the cadaver lab.  However, they did not indicate the number of specimens (full cadavers, dissected specimens etc, amount of storage solution in the storage areas (if any), size of the storage units and chambers at the experimental site.  It is suggestible to add this information in the manuscript.

 Results: The authors have mentioned that the arrangement of plants is equidistant from one another. However, did the authors observe the distance between the plants and storage areas?  Are the plants arranged equidistantly to storage places?  If so, please indicate that in the manuscript and report the correlation.

 Discussion: Requires improvement particularly on the timeline and rationale of the climatic conditions that the authors have focused on this study.  The authors have indicated that they have restricted the movement to minimize the error.  What is the permissible limit of movement if any? And did the authors notice any changes then.

Is the work clearly and accurately presented and does it cite the current literature?

Yes

If applicable, is the statistical analysis and its interpretation appropriate?

Yes

Are all the source data underlying the results available to ensure full reproducibility?

No source data required

Is the study design appropriate and is the work technically sound?

Partly

Are the conclusions drawn adequately supported by the results?

Partly

Are sufficient details of methods and analysis provided to allow replication by others?

Partly

Reviewer Expertise:

science of learning (metacognition and self-regulated learning), gamification and learning, anatomy and cytogenetics and evidence based anatomy

I confirm that I have read this submission and believe that I have an appropriate level of expertise to confirm that it is of an acceptable scientific standard, however I have significant reservations, as outlined above.

# Response

**Published:** 2024-11-22

**Authors:** Rohini Punja

**Affiliations:** Anatomy, Manipal Academy of Higher Education, Manipal, Karnataka, India

We sincerely appreciate the reviewer's thoughtful and constructive feedback, which has provided valuable insights for improving our manuscript.

 Reviewer Comment on Introduction:

 Response: We have added the details on formaldehyde composition and information about the standard concentration of formalin used for cadaver preservation in our institution, typically a 10% buffered formaldehyde solution, in the methods section. We have also specified the exact concentration of formaldehyde in the storage solution and its potential contribution to ambient formaldehyde levels.

 "In this study, we used formalin with a standard concentration of 10% formaldehyde solution, prepared according to our department's established preservation protocol. All cadavers were preserved using identical formalin preparation methods to ensure consistency in formaldehyde exposure."

 Reviewer Comment on Material and Methods:

 Response: We acknowledge the importance of providing comprehensive contextual information about the experimental environment to enhance the study's reproducibility. In the methodology section these additional details have been included:

 - Number of cadaveric specimens: Total of 15 preserved specimens

 - Storage solution details: 10% buffered formaldehyde solution

 - Storage unit dimensions: Metal preservation chambers, each approximately 2m x 1m x 1.5m

 - Total storage area volume: Approximately 21 cubic meters

 - Distance between plants: 25 cm (as originally stated)

 - Distance between plants and storage units: Approximately 50 cm

 - Plant arrangement: Centrally positioned in the storage room to ensure uniform distribution

 Reviewer Comment on Results:

 Response: We have modified the results to include specific spatial relationships and added a supplementary analysis examining potential correlations between plant placement and formaldehyde concentration variations:

 "Additional spatial analysis revealed subtle variations in formaldehyde concentration relative to plant placement. While plants were arranged equidistantly, micro-variations in ambient formaldehyde levels were observed, suggesting potential localized absorption effects that warrant further investigation."

 Reviewer Comment on Discussion:

 Response: We have attempted to improve the discussion by providing more context about seasonal conditions during the study (summer season, specific temperature and humidity ranges) and clarifying movement restriction protocols:

   * No more than 2-3 personnel entries per day

   * Maximum 5 minutes per entry

   * Strict protocol to minimize air disturbance

# Reviewer report: Reviewer response for version 1

**DOI:** 10.5256/f1000research.167255.r333509

**Published:** 2024-11-05

**Authors:** Kumar Satish Ravi

**Affiliations:** 1 All India Institute of Medical Sciences, Gorakhpur, Uttarakhand, India

Review Feedback on Manuscript

 Title: “Can indoor plants reduce formaldehyde levels in the anatomy dissection hall? A study to evaluate the practicality of using plants in reducing formaldehyde levels”

 This study presents an interesting and valuable investigation into the potential for using indoor plants to reduce formaldehyde (FA) levels in an anatomy dissection hall, a setting known for its high levels of this toxic substance. The study design, while sound, would benefit from some additional details and clarifications to provide a more comprehensive understanding of the research approach and its potential implications.

 My remarks:

 1. Description of Formalin/Preservative Used in Cadaver Preservation:

    - The manuscript could have been more informative with a clear explanation of the formalin (or other preservative) used in the preservation of cadavers. While formaldehyde is commonly used for this purpose, a brief description of the specific formulation, concentration, and the conditions under which it is used would provide additional context and clarity to the study.

    - It is important to clarify whether all cadavers in the study were preserved in the same manner and if variations in formaldehyde concentrations were considered.

 2. Methodology:

    - While the methodology is generally well structured, it could benefit from further elaboration. For example, more details about the selection process for the plant species would help readers understand why *Dracaena trifasciata*, *Epipremnum aureum*, and *Spathiphyllum* were chosen over other plants.

    - Additionally, the duration of the plant exposure (14 days) and its rationale should be more thoroughly explained. Is this duration based on prior studies, or was it selected based on practical considerations? Longer exposure periods may have yielded more meaningful results, and discussing this would strengthen the interpretation of the findings.

 3. Potential Health Concerns Regarding Plant Species:

    - A notable concern is that both *Epipremnum aureum* (Golden Pothos) and *Spathiphyllum* (Peace Lily) are known to contain calcium oxalate, which can be toxic and cause allergic reactions or skin irritation in humans. These plants are mildly toxic if ingested and can cause oral irritation, particularly in children and pets.

    - Given that these plants will be placed in an environment that involves human activity (i.e., the anatomy dissection hall), the authors should review the safety implications of using these species in such settings. This could include a discussion on the potential for allergic reactions, skin irritation, or other health concerns for individuals who may come into direct contact with the plants.

    - It is crucial to address this concern by justifying their use in the study, especially in a setting where students and faculty are working for extended hours. The authors should also explore the potential risks and whether there are safer alternatives available. A more thorough review of the literature on the toxicity of these plants in work environments would be helpful.

 4. References:

    - The manuscript cites older studies, which is acceptable to an extent, but it would be beneficial to include more recent references. The field of indoor air quality and phytoremediation is rapidly evolving, and more up-to-date studies could provide additional support for the findings or reveal emerging trends.

    - In particular, newer research on plant-based air purification, formaldehyde absorption rates, and indoor air quality in educational settings could be included to strengthen the context and relevance of the study.

 5. Statistical Analysis:

    - The statistical analysis is clearly presented, but the interpretation of the p-values and correlations could be made clearer. Specifically, the discussion on the weak positive correlation with formaldehyde levels should be more thoroughly addressed. Given the results, the authors could explore the reasons why certain plant species did not show a significant reduction in FA levels, despite previous studies suggesting their effectiveness. This could involve discussing the environmental factors (e.g., temperature, humidity) or other variables not accounted for in the study.

 6. Conclusion and Future Research:

    - The conclusion is well-written and appropriately acknowledges the limitations of the current study. However, it would be beneficial to provide more specific recommendations for future research. For instance, the authors could suggest conducting a follow-up study with a larger sample of plants or extended exposure periods to better assess the long-term effectiveness of indoor plants in reducing formaldehyde levels.

    - Additionally, exploring how different plant species interact with environmental variables (e.g., light, humidity, temperature) would help refine the understanding of which plants are most effective for reducing air pollutants in real-world settings.

Is the work clearly and accurately presented and does it cite the current literature?

Yes

If applicable, is the statistical analysis and its interpretation appropriate?

I cannot comment. A qualified statistician is required.

Are all the source data underlying the results available to ensure full reproducibility?

Yes

Is the study design appropriate and is the work technically sound?

Yes

Are the conclusions drawn adequately supported by the results?

Yes

Are sufficient details of methods and analysis provided to allow replication by others?

Yes

Reviewer Expertise:

Anatomy, Cytogentics, Medical Education, Neuroanatomy

I confirm that I have read this submission and believe that I have an appropriate level of expertise to confirm that it is of an acceptable scientific standard, however I have significant reservations, as outlined above.

# Response

**Published:** 2024-11-22

**Authors:** Rohini Punja

**Affiliations:** Anatomy, Manipal Academy of Higher Education, Manipal, Karnataka, India

We sincerely thank you for the constructive feedback and have tried to address the concerns as pointed. 

 1. Description of Formalin/Preservative:

 Response: We have added a more detailed explanation of the formalin used in cadaver preservation in the Methods section. We have specified the concentration, formulation, and standard preservation conditions used in our anatomy department. We have also added that all cadavers were preserved using the same standardized protocol.

 "In this study, we used formalin with a standard concentration of 10% formaldehyde solution, prepared according to our department's established preservation protocol. "All cadavers were preserved using identical formalin preparation methods to ensure consistency in formaldehyde exposure."

 2. Methodology:

 Response: We appreciate the feedback on our methodology. We have justified the rationale for selecting *Dracaena trifasciata (Sansevieria), Epipremnum aureum*, and *Spathiphyllum*, including their previously reported air purification capabilities.The duration of 14-day exposure period was due to time constraints and this has been included explaining our practical considerations. We have discussed the potential benefits of longer exposure periods in the limitations and future research sections.

 "Plant Selection Rationale: "We selected these three plant species based on their prior reported capabilities in air purification. *Sansevieria trifasciata *(Snake Plant), *Epipremnum aureum* (Golden Pothos), and *Spathiphyllum* (Peace Lily) have been previously documented in scientific literature for their potential to absorb volatile organic compounds."

 3. Potential Health Concerns Regarding Plant Species:

 Response: We acknowledge the important safety considerations raised. We have included in the discussion the potential toxicity of *Epipremnum aureum* and *Spathiphyllum*, explaining the ideal placement of plants to minimize direct human contact. Since this was conducted in the specimen storage room and students do not have access to this area, except the staff handling these specimens/cadavers the risk of exposure is minimum though we have to keep these points in mind if we plan to replicate this study in an environment where students would be present.

 4.References:

 Response: We have updated our references with more recent studies on indoor air quality and phytoremediation and included newer research on plant-based air purification, particularly focusing on studies conducted in educational settings after 2018.

 17. Matheson S, Fleck R, Irga PJ, Torpy FR. Phytoremediation for the indoor environment: a state-of-the-art review. Rev Environ Sci Biotechnol. 2023;22(1):249-280. doi: 10.1007/s11157-023-09644-5. Epub 2023 Feb 27. PMID: 36873270; PMCID: PMC9968648.

 18. Peterson G, Jones T, Rispoli D, Haddadi S, Niri V. Investigation of simultaneous volatile organic compound removal by indoor plants using solid phase microextraction-gas chromatography-mass spectrometry. RSC Adv. 2023 Sep 7;13(38):26896-26906. doi: 10.1039/d3ra04015a. PMID: 37692358; PMCID: PMC10483372.

 5. Statistical Analysis:

 Response: We have provided a more detailed interpretation of the weak positive correlations and discussed potential environmental factors that might have influenced plant performance and also explored why our results differed from previous studies, considering variables like temperature, humidity, and room configuration

 "The weak positive correlation observed might be attributed to complex environmental interactions. "Factors such as room temperature (ranging from 26.3-33.4°C), humidity variations (67-85%), and specific room configuration could significantly influence plant performance in formaldehyde absorption."

 6. Conclusion and Future Research:

 Response: We have expanded our conclusion to include more specific recommendations for future research; suggestions for follow-up studies with larger plant samples and extended exposure periods and exploration of how environmental variables interact with plant performance in air purification
